# Exploring the career choices of White and Black, Asian and Minority Ethnic women pharmacists: a qualitative study

**DOI:** 10.1111/ijpp.12424

**Published:** 2017-12-26

**Authors:** Kelly Howells, Peter Bower, Karen Hassell

**Affiliations:** ^1^ NIHR School for Primary Care Research The University of Manchester Manchester UK; ^2^ College of Pharmacy California North State University Elk Grove CA USA

**Keywords:** pharmacy workforce, employment choices, women, ethnic minorities, qualitative

## Abstract

**Objective:**

In the UK, a growing number of females entering pharmacy are women from Black, Asian and minority ethnic groups (BAME). Research shows that BAME women are more likely to work in the community sector and be self‐employed locums than white women, and Asian women overrepresented in part‐time, lower status roles. This study aims to explore the employment choices of white and BAME women pharmacists to see whether their diverse work patterns are the product of individual choices or other organisational factors.

**Methods:**

This study analyses 28 qualitative interviews conducted with 18 BAME and 10 white women pharmacists. The interview schedule was designed to explore early career choices, future career aspirations and key stages in making their career decisions.

**Key findings:**

The findings show that white and BAME women are influenced by different factors in their early career choices. Cultural preferences for self‐employment and business opportunities discourage BAME women from hospital sector jobs early in their careers. Resonating with other studies, the findings show that white and BAME women face similar barriers to career progression if they work part‐time.

**Conclusions:**

Women working part‐time are more likely to face workforce barriers, irrespective of ethnic origin. Cultural preferences may be preventing BAME women from entering the hospital sector. This research is important in the light of current debates about the future shape of pharmacy practice, as well as wider government policy objectives that seek to improve the working lives of health care professionals and promote racial diversity and equality in the workplace.

## Introduction

The demographic profile of the pharmacy profession has significantly changed. In 1964, 19% of registered pharmacists were female. Recent register data (2017) show that the majority of registered pharmacists in Great Britain (GB) are female (61.9%),^1^, ^2^, ^3^ as well as in the United States of America (USA)^4^ and Canada.^5^ The ethnic profile of the profession in GB has also changed significantly. In 1975, Black, Asian and Minority Ethnic (BAME) groups accounted for 15% of registered pharmacists. In 2017, 49% of registered pharmacists who reported their ethnic origin (49 520/55 209) were from a BAME group. The majority of BAME pharmacists identify themselves as Asian with Indian Asians being predominant.^1^, ^2^, ^6^

While the growing representation of both women and ethnic minorities in pharmacy may be an example of occupational integration,^7^, ^8^ women pharmacists’ employment patterns continue to mirror the gendered division of labour.^6^, ^9^ There is debate as for whether the persistence of this gender dichotomy in pharmacy and elsewhere is linked to gendered employment choices, or organisational and structural constraints.^3^, ^7^, ^8^, ^10^, ^11^, ^12^, ^13^, ^14^, ^15^, ^16^

Pharmacy is also segmented along ethnic lines, with BAME groups, particularly Indian pharmacists, over‐represented in the community sector and as self‐employed contractors.^6^ Studies have identified personal preferences for business ownership as largely responsible.^17^, ^18^, ^19^ While a cultural bias for self‐employment in Asian cultures may be underpinned by a preference to avoid discrimination and increase occupational mobility,^19^ there is limited evidence that ethnic‐based discrimination is a factor in pharmacy as in other professions such as medicine.^20^

Empirical work to date has focused little attention on the position of BAME women pharmacists. In the past, ethnic differences between women have largely been unexplored due to small sample sizes^21^ This is no longer the case; 45% of women pharmacists are now from a BAME group.^1^ In urban areas such as Manchester, the majority of women pharmacists are from a BAME group (59%)^1^ The statistics suggest that irrespective of ethnic origin, gender divisions persist in pharmacy.^[8,21]^ The employment choices of White and BAME women are not homogeneous, with White women being significantly more likely to work part‐time than BAME women. BAME women, such as men pharmacists, are also under‐represented in hospital pharmacy and are more likely to work in the community.^22^

While gender segregation is prevalent in both the community and hospital sector, research shows that when women mirror the work patterns of men and work full‐time, they are more likely to ‘break the glass ceiling’.^23^, ^24^ However, the employment patterns of BAME women pharmacists highlight an important paradox; BAME women are significantly more likely to work full‐time than their White counterparts, but are significantly less likely to occupy management positions.^21^, ^25^ However, this finding was reported at a time when analysis of individual ethnic groups was not possible. Analysing BAME women pharmacists as a homogeneous group is misleading as not all BAME women are under‐represented in management positions.^22^ In community pharmacy, Black and Chinese women (irrespective of dependent status) are significantly more likely to occupy management positions than White and Asian women.^2^ Asian and White women pharmacists are similarly likely to work part‐time, indicating that the polarised employment positions of women in community pharmacy are linked to the hours they work rather than ethnic origin. In the hospital sector, BAME women are less likely than White women to occupy management positions and are almost three times as likely to be self‐employed locums. Although age could be a factor, as the mean age of BAME women is younger than White women,^2^ what is not known is why BAME women are more likely to work in the community sector and be self‐employed than White women.

Research on this topic is important in the light of current debates about the future shape of pharmacy practice.^26^, ^27^ With an ageing population, the pharmacy profession is evolving to meet the increased demands. A collaborative care approach is high on the agenda, with clinical pharmacists in GB working in primary care to support GPs with prescribing. The role of community pharmacists is also evolving with more undertaking training as independent prescribers. As at least half of all women pharmacists prefer to work part‐time and not engage with career development opportunities,^7^, ^8^, ^13^ there could be concern that women pharmacists, particularly Asian women, who are more likely to both locum and work in the community sector part‐time, are less likely to ‘up‐skill’ than their male counterparts.

The aim of the study was to understand if the career choices of White and BAME women pharmacists are influenced by different factors. The objectives of the study were

- To explore if the career choices of White and BAME women pharmacists were the result of individual agency or structural constraints
- To explore if BAME women pharmacists face different barriers to career progression and development than White women pharmacists

## Methods

A qualitative approach was used to address the study objectives. Semi‐structured interviews were conducted with registered women pharmacists currently working in the community or hospital sector. There were no other inclusion criteria. The sampling strategy was opportunistic and relied on convenience sampling. Participants were recruited by pharmacists already known to the researcher. Following the initial interviews, the researcher asked participants if they could recommend other women pharmacists to be interviewed.

Eligible participants received an information sheet with an interview schedule prior to the interview and were asked to return a signed consent form To ensure women from a range of different ethnic backgrounds were sampled, participants were also asked to complete a short questionnaire to collect demographic information. Ethnic origin was determined using the classification in the 2001 UK census.

The interviews took place either in the participant's workplace or home. The number of interviews conducted was to be based on theoretical saturation. Ethical approval was obtained from an NHS Research Ethics Committee (Ref 09/H10114 ‐22/01/2009).

A White female researcher conducted all the interviews. An interview schedule was developed initially from the research literature, with some changes added after piloting. Influenced by a socio‐biographic approach,^28^, ^29^ all the interviews began in the same manner, with a life history of why they chose to study pharmacy, their training experiences, their initial pharmacy post and future aspirations. Participants were also asked if they felt they had experienced any form of professional discrimination.

The interviews were analysed using a deductive approach, whereby, the analysis of qualitative data is guided by existing theoretical evidence; if the evidence contradicts existing theory a new theory can be developed.^30^, ^31^ The analysis was guided by McRae's concept of normative and structural constraints.^32^ Using NVIVO, potential relationships between the concepts were identified and compared to assess which factors were most important in explaining the employment choices of White and BAME women pharmacists. Transcripts were coded by the research team, who met monthly to discuss emerging themes.

## Results

A total of 28 semi‐structured interviews were completed with 10 White and 18 BAME women pharmacists (see Table 1) in Greater Manchester, England. Demographic data are provided in Tables 1 and 2. All interviews were audio‐recorded and transcribed verbatim (duration was 28–62 min). Only the interviewer and participant were present during the interviews.

**Table 1:** Summary of participant characteristics

Hours worked	Community	Hospital	Number of participants with children under 18
Part‐time (<30 h)	7	3	9
Full‐time	11	7	4
Total	18	10	13
Employment position			
Locum	6	1	1
Owner	1	0	1
Manager (Grade 8 or above)	4	5	4
Employee (Grade 7 or below)	7	4	7
Total	18	10	13
Ethnic origin			
White	7	3	
Black African/ Caribbean	1	2	
Pakistani	5	1	
Indian	3	1	
Asian other	0	1	
Mixed race	1	0	
Chinese	1	0	
Middle Eastern	0	2	

**Table 2:** Summary of age and grade

Age	Locum	Practitioner/grade 6 and 7	Manager/owner/Grade 8	Totals
21–30	4	3	2	9
31–40	2	8	5	15
41–50	0	0	3	2
50–60	1	0	0	1
60+	0	0	0	0
Total	7	11	10	28

The results are presented under the three main themes: early career choices, career preferences and constraints on career choices. Verbatim quotes will be used to illustrate the key themes in text boxes. Quotes will be identifiable by italicised text, anonymised interview ID and participant's key characteristics.

### Early career choices

Almost half of the BAME women mentioned an initial preference for medicine over pharmacy compared with *none of the* White women interviewed (see Textbox 1).

Textbox 1 **Quotations representing early career choices theme**

‘If you do know anything about Eastern culture, everybody wants to be a doctor, so I'm no different. I applied for medicine, number 1 choice, 2, pharmacy and 3, dentistry and was accepted in dentistry and pharmacy’ (P19, British Middle Eastern, one child).

‘I was looking at what I could do with my options and I wanted to do something ‘sciency’ and then I thought, pharmacy, and I thought well I like the topics that are covered on the degree course so I thought I may as well as do that’ (P6 White British, no children).

‘I was good at sciences and I wanted a professional, like reputable profession to go into that would guarantee me more or less a job’ (P3 British Pakistani, no children).

‘I don't think I mentioned at the beginning but whilst I was thinking about doing my career, a family is important to me because of my culture…I'm Hindu, yes, so because it plays such an important part of our culture and I've taken that on board I did have to kind of think about well in the future I'm going to have kids, I want to be around and there for them’ (P27, British Indian, no children)

‘It's very much an Asian thing to have your own business… And you know it's the perception of what a pharmacist is…that you are a business person really, I think.’ (P9 British Pakistani)

Self‐employment was held in high regard culturally by some of the Asian pharmacists interviewed, and opportunities to locum were considered beneficial once they had completed their training (see Textbox 1). All the locums interviewed (*n* = 7) were from a BAME group. Family influences were an important factor in shaping their perceptions of a pharmacy career as a ‘business’, so a career in the hospital sector was rarely considered.

Job security and the initial higher earnings were stated by both White and BAME women community pharmacists as an integral reason for their pharmacy sector choice. In hospital pharmacy, the additional qualifications needed to climb the career ladder deterred some BAME and White women from applying as they wanted to start earning money ‘sooner rather than later’.

Both White and BAME women (across all age groups) also perceived pharmacy to be ‘a good job for a woman’, due to the perceived flexible working ethos in the profession (See Textbox 1). Participants with and without children were attracted to the profession for this reason, and this career preference had deterred some of the BAME women from applying for medicine.

### Career preferences

The participants working part‐time were unanimous in their view that part‐time working was not a compromise, but a choice made within the context of what they perceived to be ‘a good mother’ (See Textbox 2). Irrespective of ethnic origin, all the part‐time workers conveyed similar motherhood ideologies and preferred to ‘spend time’ with their children rather than prioritise career progression. For some participants, it was inconceivable that they could progress to management on a part‐time basis. However, this was viewed as a personal rather than constrained choice.

Textbox 2 **Quotations representing career aspirations**

‘I took a year off for both of them and I just didn't like the idea of leaving them in a nursery for five days a week, I wanted to spend some time with them and I think the first years, all years of your life are important but the first few years are so precious… Just to leave them at nursery just to be cared for my someone else, I wanted to do that myself, so working two or three days is just ideal’ (P14, British Indian community pharmacist, 2 children)

‘When I had my first child I knew I wanted to spend lots of time with him, I didn't want to, I didn't want him to go to nursery and me spend most of my time working, so it was a choice.’ (P24, White British community pharmacist)

‘I can apply for band 7, a lot of people still apply whilst they are still doing the diploma. I've not really gone for a band 7 for a number of reasons. There's been quite a few positions come up here but nothing that I'm really interested in, and then I got married last year, been pregnant so that's kind of put my career to the back of my head, it's not really important anymore.’ (P1, British Middle Eastern, grade 6 hospital pharmacist, pregnant).

‘It's not that I'm so averse to being a manager and a mum but I don't feel that it's necessarily fair in that situation…I don't think that I could commit personally, could commit enough, provide the staff with what they need. I don't think it would be the company telling me that I couldn't do it but I think it would be more my choice that I wouldn't want to take it on’ (P22, White British pharmacy manager, no children).

P26 and P15 had previously worked part‐time when their children were younger, for both moral and practical reasons. P26 argued that it was ‘impossible to work full time and juggle childcare’ so she had compromised by working part‐time.

Participants without children admitted that their career aspirations may change in the future and they would most likely reduce their hours and downgrade their roles to maintain a work‐life balance. There were no ethnic differences with respect to this view (see Textbox 2)

Three participants with children under age 10 worked full‐time. Although for two participants, their working hours reflected their career aspirations (P23 and P2) for one participant (P19) her choice was motivated by financial necessity rather than personal preference.

### Constraints on employment choices

Managers were pivotal in both enabling and constraining career choices.

Management support enabled some participants to fulfil their working time preferences (P8 and P20 – see Textbox 2). There was, however, a lack of homogeneity with regard to how flexible working opportunities were implemented across the profession. P8 for example was given a term time contract, but was quick to point out that ‘(the organisation) keep this fact quiet because otherwise everyone would want one’.

Flexible working was considered an important factor in enabling upward career mobility. P14 (British Indian, part‐time community pharmacists), and P18 (British Black African, part‐time hospital pharmacist), both had to downgrade their roles on returning to work part‐time following maternity leave (see Textbox 3). P18 was working in the community sector and changed to the hospital to enable her to move up the career ladder on a part‐time basis. Although job shares are available in community pharmacy, there was little support for this way of working and there hence appeared to be a disconnect between company policy and implementation.

Textbox 3 **Quotations representing career constraints**

‘I only went term time last year and that was because both my parents died last year… You can only rely so much on friends, but you can't rely on friends 6 weeks in the summer holidays to look after your child for 4 days a week so that was when I asked about these term time (contracts), actually it was suggested to me I didn't actually think (the pharmacy) did term time contracts anymore, but they do. But I think they keep them a bit quiet because otherwise everyone would want one so because it was exceptional circumstances really it was offered to me’ (P8, White British, part time, second community pharmacist, one child)

‘When I came back I did want a job share, but I was told that the company were not supporting job shares, I'd heard. I wasn't officially told, erm, just a few people I knew had said that as a company, they are not supporting job shares’ (P14, British Indian, part time second community pharmacists, two children).

‘I was in community, then got married and had a child and that was one of the reasons I left community… it's so much harder to be in community and have children… When your child is ill for instance, and you have no family near you, because my family don't live here, they live abroad and my husband's family don't live anywhere near so we have absolutely no support. And when a little kid is ill and the nursery phones you to leave obviously you cannot leave the shop because that's impossible, because you'll have to close down the shop (P15, Full tome Hospital Pharmacists, White British)

‘I went back from maternity leave, from my original job in London, and the head of department said, you can't be a part time manager.’ (P18 British Black Caribbean, part time, 2 children)

‘I wouldn't want to be contracted to them…I prefer being a locum and having my independence’ (P4 British Indian community pharmacy locum, 1 child)’

‘There are things like the clinical diplomas and things like that, that I would have loved to have pursued but don't suppose I work enough to justify them.. You know you feel you are not going to justify it with how many hours I work at the moment’ (P12 White British, part‐time, 4 children, married).

Changing pharmacy sector to secure flexible working was not uncommon, (See Textbox 3). For those women wanting to remain in the community sector, they often decided early on in their careers to become a locum pharmacist. In total, seven locums, (all BAME women), the majority of locums interviewed (6 of 7) did not have children but preferred the flexibility and high pay associated with locum work. It was for this reason the locums interviewed stayed in the community sector, as locum work is not widely available in the hospital sector.

Although some participants were frustrated with the fewer opportunities for career development and progression, they generally accepted this predicament as a ‘trade‐off’ to work part‐time (see Textbox 3).

### Perceptions of discrimination

While women working part‐time had experienced limited access to training and downward career mobility (see Textbox 4), these constraints were not recognised as discrimination by the women interviewed. Gender discrimination was normalised in this context as something that was an inevitable and understandable consequence of working part‐time (See Textbox 3).

Textbox 4 **Perceptions of discrimination**

‘Well I was ready for a management position much earlier than I got one…I felt that the area manager at that time wasn't taking me seriously, but when the area manager changed, and another very good brown faced friend of mine put a good word in for me and that's how I feel I got my first management position faster… somebody I knew, who was also a brown face, who was high up and was in with that ‘crowd’ put a good word in for me and that's how I got my first management position’ (P13, British Pakistani).

‘He (the customer) said ‘since you've been here, you're not even from this country, since you have been here the pharmacy is going down…in front of the shop, and nobody said anything… my team members are standing there they didn't say anything… no nobody, not a single person, and people were actually avoiding eye contact with me and I thought I can't believe you can actually stand here and watch me be racially abused’ (P21, British Black African).

‘Because at that time I'd come from Pakistan and wasn't very fluent in English I was speaking, I was trying. I think she had a problem with me trying to say things lots of time, it took longer than a normal person to finish a sentence because I had all the words in my mind and I had to pronounce them in a certain way and it wasn't happening. Because I had to put lots of effort into getting certified, as I had to prove I was as good as a native English speaker but maybe sometimes you have to bear with people sometimes…I used to come home and cry myself to sleep because she used to make me scared to go to work…she was my tutor and I was a bit angry when I finished…I didn't know back then that I could actually complain about her’ (P17, British Pakistani).

In discussing ethnic‐based discrimination, the majority of participants had not experienced it. However, P1 (Black African) and P13 (Pakistani) both indicated that career progression was not based on meritocracy but rather by ‘who you know’. P13 felt that her senior colleagues were always part of the ‘same crowd’ and she felt as a Muslim woman, she was at a disadvantage as she couldn't necessarily network with senior colleagues socially, as they often drank alcohol. Although this participant had not experienced any form of direct discrimination, she felt discouraged from seeking promotion because of the predominately White hierarchy in the hospital sector (see Textbox 4). P13 also indicated that her progression into management had been made possible by a fellow Asian woman pharmacy manager ‘who was in the crowd’ and therefore made it easier for her to progress (see Textbox 4). Some participants (P21 and P17) had experienced bullying and harassment from patients and colleagues.

As illustrated in the quotes, there was a lack of collegial support and neither participant felt they would complain. A culture of support, whereby equal opportunities are promoted, and all staff are reminded of ways in which they can complain about work problems, is thus essential in maintaining an organisational culture which promotes gender and ethnic equality.

## Discussion

This is the first published study exploring the career choices of White and BAME women pharmacists. The findings highlight that irrespective of ethnic origin, White and BAME women will encounter similar barriers to career progression if they work part‐time. White and BAME women working part‐time had similar career aspirations. The analysis suggests that normative factors (such as cultural ideals and parental expectations about medical and pharmacy careers) were critical influences on BAME women's pharmacy sector preferences. Cultural preferences help explain why community pharmacy is the sector of choice for the majority of BAME women in the profession, particularly as the self‐employment opportunities associated with the community sector are deemed suitable for a woman with children, particularly younger children.

### Limitations

Non‐response bias is a possible limitation of the study. Women who have experienced more direct discrimination may not have wanted to participate, particularly as the ethnic origin of the researcher (White British) may have deterred them. The researcher acknowledges that BAME women may feel more comfortable discussing discrimination with someone from a BAME group. Theoretical saturation was also not achieved as the research themes could not be explored in further detail due to the insufficient number of BAME women in the hospital sector (there were four local hospital pharmacies compared to 132 community pharmacies). We were also unable to interview women pharmacists who had left the profession and this may have provided further insight into preferred working practices. Interview participants all lived in the Manchester area and the majority of interviewees were from a BAME group, so the sample was not representative of GB in general. Women in rural areas, which are less ethnically diverse, may have a different experience.

### Results

Findings both support and extend the existing literature in medicine and pharmacy which have shown a relationship between career progression and hours worked.^10^, ^12^, ^13^, ^14^, ^33^

Resonating with previous research findings, women part‐time workers had broadly similar career trajectories and opportunities irrespective of ethnic origin.^4^, ^12^, ^14^, ^15^, ^16^, ^34^ There was some evidence that cultural perceptions of motherhood underpinned women's working time preferences. This was more prominent in the interviews with the White, Asian and Middle Eastern women interviewed. Women without children also hinted at working part‐time in the future. However, one of the Black women interviewed had previously worked part‐time when her children were younger, showing that women should not always be pigeon‐holed because of their ethnic origin and a variety of other factors, such as their children's age, social context, partner status and financial situation are also significant in shaping the career choices of women.

The women working full‐time had not encountered gender discrimination, but they did express concern about remaining in management roles after children. While this concern was to an extent a reflection of their own preferences, there seemed to be an acceptance that management roles were incompatible with part‐time working. These concerns were validated by participants who had experienced downward career mobility on returning to work part‐time. While women part‐time workers had fewer opportunities for career progression or training, indirect gender discrimination was normalised as inevitable. As White and Asian women are more likely to work part‐time than women from Black and other minority ethnic groups, they are therefore more likely to encounter indirect gender discrimination.

Cultural ideals relating to self‐employment continued to underpin the career choices of some BAME women later in their careers. All the locums interviewed were BAME women who valued the flexible working patterns and high pay. This, however, came at a cost as locums are generally unable to participate in career development opportunities, (such as the clinical diploma), and workplace pensions. As BAME women are significantly more likely to locum than their White counterparts. BAME women may, therefore, be disproportionately disadvantaged long‐term compared to White women in making this career choice.

Resonating with other research,^35^, ^36^ there was limited evidence that perceptions of ethnic discrimination may affect the career decisions of BAME women pharmacists. A number of participants implied that having a manager of the same ethnic origin would have a positive effect on career development. The lack of BAME women mentors, particularly in hospitals, in addition to cultural perceptions of pharmacy, may deter some BAME women from entering hospitals and climbing the career ladder in this sector. For employers, positive action recruitment strategies may be useful in this context to encourage more BAME women to apply for positions in the hospital sector. Universities should also consider how they encourage BAME women to consider a career in the hospital sector.

## Conclusion

Reflecting on the research aims, the analysis highlights that irrespective of ethnic origin, organisational factors made career progression and development difficult for some women, particularly women working part‐time. While some women were able to overcome these barriers due to family and management support, others remain segregated in lower status roles to maintain a work‐life balance.

While there was limited evidence that BAME women are more likely to perceive that they have experienced barriers to career progression than White women, pharmacy sector choice was important in understanding their career choices. There was limited evidence that the lower number of BAME women in the hospital sector is related to discrimination. However, the low number of BAME women in the hospital sector combined with cultural preferences for self‐employment and business opportunities may be preventing BAME women from applying to this sector early on in their career.

The organisational structures in the community sector are considered to be more gendered than in the hospital, so unless women community pharmacists are prepared to work full‐time, they may find more opportunities for occupational mobility in the hospital. This may be because of the linear and meritocratic career structures in the hospital sector, which may make it easier for women to progress than the culture of long hours in the community. As Asian women are more likely than other women to work in the community and part‐time, Asian women may be more likely to encounter indirect gender discrimination than other women pharmacists.

## Declarations

### Conflict of interest

The Author(s) declare(s) that they have no conflicts of interest to disclose.

### Funding

This study was funded by The University of Manchester.

### Authors’ contributions

All Authors contributions adhere to the International Committee of Medical Journal Editors’ definition of authorship.KH and KH contributed to the conception and design of the study. Kelly Howells collected the data. KH and KH contributed to the analysis of the data. All authors contributed to the interpretation of the data. Kelly Howells led on drafting the article. All authors contributed to drafting and revising the content critically.
